# The Bipolar Depression Electrical Treatment Trial (BETTER): Design, Rationale, and Objectives of a Randomized, Sham-Controlled Trial and Data from the Pilot Study Phase

**DOI:** 10.1155/2015/684025

**Published:** 2015-03-24

**Authors:** Bernardo de Sampaio Pereira Junior, Gabriel Tortella, Beny Lafer, Paula Nunes, Isabela Martins Benseñor, Paulo Andrade Lotufo, Rodrigo Machado-Vieira, André R. Brunoni

**Affiliations:** ^1^Center for Clinical and Epidemiological Research & Interdisciplinary Center for Applied Neuromodulation (CINA), University Hospital, University of São Paulo, São Paulo, Brazil; ^2^Service of Interdisciplinary Neuromodulation (SIN), Department and Institute of Psychiatry, Faculty of Medicine of University of São Paulo, São Paulo, Brazil; ^3^Laboratory of Neuroscience (LIM27), Department and Institute of Psychiatry, University of São Paulo, São Paulo, Brazil; ^4^Bipolar Disorder Research Program, Department and Institute of Psychiatry, University of São Paulo Medical School, Brazil; ^5^Experimental Therapeutics and Pathophysiology Branch, Intramural Research Program, National Institute of Mental Health (NIMH), NIH, Bethesda, MD, USA; ^6^Hospital Universitário, Cidade Universitária, Universidade de São Paulo, Avenida Professor Lineu Prestes 2565, No. 3 Andar, 05508-000 São Paulo, SP, Brazil

## Abstract

*Background*. Bipolar depression (BD) is a prevalent condition, with poor therapeutic options and a high degree of refractoriness. This justifies the development of novel treatment strategies, such as transcranial direct current stimulation (tDCS) that showed promising results in unipolar depression. *Methods*. We describe a randomized, sham-controlled, double-blinded trial using tDCS for refractory, acutely symptomatic BD (the bipolar depression electrical treatment trial, BETTER). Sixty patients will be enrolled and assessed with clinical and neuropsychological tests. The primary outcome is change (over time and across groups) in the scores of the Hamilton Depression Rating Scale (17 items). Biological markers such as blood neurotrophins and interleukins, genetic polymorphisms, heart rate variability, and motor cortical excitability will be assessed. Twelve anodal-left/cathodal-right 2 mA tDCS sessions over the dorsolateral prefrontal cortex will be performed in 6 weeks. *Results*. In the pilot phase, five patients received active tDCS and were double-blindly assessed, two presenting clinical response. TDCS was well-tolerated, with no changes in cognitive scores. *Conclusion*. This upcoming clinical trial will address the efficacy of tDCS for BD on different degrees of refractoriness. The evaluation of biological markers will also help in understanding the pathophysiology of BD and the mechanisms of action of tDCS.

## 1. Introduction

Bipolar disorder is a highly prevalent condition, with poor symptomatic and psychosocial outcomes [1]. It is characterized by episodes of mania—when the patient experiences racing thoughts, elated mood, grandiosity, and low necessity for sleeping—and depression—characterized by sadness, thoughts of guilt, and incapacity in feeling pleasure and interest in daily activities once considered joyful. Depressive episodes in bipolar disorder are, in fact, more prevalent and might be more disabling than the manic episodes [2, 3]. Moreover, the treatment of bipolar depression (BD) is limited to a few effective drugs, which present important side effects, such as cognitive impairment [4] and metabolic side-effects, leading to nonadherence and treatment discontinuation [5]. Guidelines also diverge regarding the optimal treatment: whereas some recommend only lithium, lamotrigine, and quetiapine as a first-line treatment [6], others allow the use of antidepressant drugs [7], which should be used in association with mood stabilizers due to the risk of treatment-emergent (hypo)mania, and other anticonvulsants and antipsychotics [8]. For refractory BD, the available level I evidence is very scarce, with only five studies exploring this issue hitherto [9]. Considering novel therapies, in two controlled trials, ketamine was superior to placebo but the effects were short-lived; in addition, ketamine is not orally available; pramipexole was barely superior to placebo in one controlled trial; and three other drug trials were not significant versus placebo [10]. This reinforces the need of novel and more efficacious treatments for refractory bipolar depression.

In this context, noninvasive brain stimulation therapies have been increasingly investigated as a treatment for neuropsychiatric disorders, particularly mood disorders [11]. One of them is repetitive transcranial magnetic stimulation (rTMS), which is effective, for instance, in treatment-resistant unipolar disorder [12], although few studies have addressed rTMS efficacy in BD, with mixed results; for instance, an early double-blinded, sham-controlled trial in 23 patients [13] failed to show positive results, whereas Dell'Osso et al. [14] demonstrated rTMS efficacy in an open-label study with 11 patients, and, likewise, Cohen et al. [15] found positive results in a naturalistic, follow-up study with 56 patients.

Another nonpharmacological intervention is transcranial direct current stimulation (tDCS), a brain stimulation technique that presents low cost, high portability, benign profile of adverse effects and it is relatively simple to use [16, 17]. TDCS consists in applying a weak, direct current through two electrodes placed over the scalp, the anode and the cathode increasing and decreasing cortical excitability during and beyond the period of stimulation [18]. Trials investigating the potential therapeutic effects of tDCS for treating major depressive disorder (MDD) showed promising results. For instance, Loo et al., in a randomized, sham-controlled trial, evaluated 64 depressed patients, finding a greater improvement in depressive symptoms after active versus sham groups, although no difference in responder rates (13% for both groups) was found. Brunoni et al., in another sham-controlled design recruiting 120 patients [19], reported that tDCS and sertraline 50 mg/day were similarly effective in patients with MDD, the combined treatment leading to increased effects. A recent meta-analysis [20] enrolled seven randomized, sham-controlled tDCS depression trials (*n* = 259). Although most studies were heterogonous and enrolled relatively small sample sizes, active versus sham tDCS was significantly superior for all outcomes (response, remission, and changes in depression scores).

However, although the field of tDCS as a MDD treatment significantly advances, the efficacy of tDCS for BD has been insufficiently investigated—studies which are limited to depression trials in which BD patients were also enrolled—such as in the study of Loo et al. [21], in which eight patients with BD (four in each group) were recruited, and an open-label study that compared tDCS effects in patients with unipolar versus bipolar depression [22]. Both studies suggested that tDCS has antidepressant effects in BD, although no study was specifically designed for prospectively assessing the efficacy of tDCS in BD.

Therefore, considering the need to develop novel strategies for the treatment of BD and the encouraging findings of the antidepressants effects of tDCS in unipolar depression, our aim is to address the antidepressant effects of tDCS in refractory BD in a randomized, sham-controlled trial. Here, we describe the design of this study and also provide data of 5 refractory BD patients treated with tDCS during the pilot phase of our study.

## 2. Methods

### 2.1. Participants

The BETTER (bipolar depression electrical treatment trial) study will enroll 60 adults aging from 18 to 65 years diagnosed with bipolar disorder (type I or II or not otherwise specified) in an acute depressive episode according to DSM-5 (Diagnostic and Statistical Manual of Mental Disorders, 5th edition) criteria and confirmed by the Mini-International Neuropsychiatric Interview (M.I.N.I.). Demographic and clinical data will be collected, including age, age at onset of the first episode, marital status, occupational status, diagnosis subtype, duration of illness, number of failed antidepressant treatments during the current episode, and family history for mental disorders in first-degree relatives. Since, at the present moment, there is no structured questionnaire available specifically tailored for the new DSM-V criteria, we will use the MINI (based on DSM-IV-TR) with some adjustments as to assess changes in diagnostic criteria that were done in DSM-5; for example, mixed episodes no longer constitute an independent category but instead may occur during episodes of (hypo)mania or major depression. Refractoriness will be assessed using the Antidepressant Treatment History Form (ATHF) and according to CANMAT 2013 guidelines, which defined first- and second-line treatments for BD [6], and also according to the systematic review of Sienaert et al. systematic review [10] that considered refractoriness as failure to improve after at least two treatment strategies, of which at least one was a mood stabilizer.

Eligibility criteria include the presence of a depressive episode of at least moderate intensity, corresponding to a Hamilton Depression Rating Scale (17-items) (HDRS-17) > 17. Exclusion criteria are (1) other neuropsychiatric conditions, such as schizophrenia, substance dependence, dementias, traumatic brain injury, epilepsy, personality disorders, and anxiety disorders (although participants with the two latter disorders can be included whether the primary diagnosis is BD); (2) simultaneous presence of (hypo)manic symptoms, indexed by a Young Manic Rating Scale (YMRS) > 8; (3) bipolar disorder patients who have rapid cycling; (4) acute suicidality; (5) pregnancy; (6) specific contraindications to tDCS, such as electronic or metal implants in the cephalic segment previous participation in other tDCS trials; and (7) severe/life-threatening clinical conditions. Participants will have to be either drug-free or at stable drug regimen for at least two and four weeks for mood stabilizers and antidepressant drugs, respectively. Benzodiazepine drugs will be allowed, although only at low doses (less than 20 mg/day of diazepam or equivalent).

Recruitment strategies include a convenience sample of depressed patients from an outpatient ambulatory, referred and screened by psychiatrists specialized in mood disorders and spontaneous demand through advertising in local newspapers, radio stations, and websites.

### 2.2. Interventions

For tDCS, the anode is placed over the left dorsolateral prefrontal cortex that is located in F3 (according to the International EEG System 10–20) and the cathode electrode over the right dorsolateral prefrontal cortex (F4), similar positioning that was firstly proposed in Ferrucci et al. study [23]. The rubber electrodes are involved in 25 cm^2^ saline-soaked sponges and fixed with a headband. We use a direct current of 2 mA (current density = 0.80 A/m^2^) for 30 min per 10 consecutive workdays followed by one session every other week, for 4 weeks (total charge density of 1440 C/m^2^), a similar design used by our group in the SELECT-TDCS [24].

For sham conditions, the device (Soterix Medical, New York, NY, USA) is turned off after 30 seconds of active stimulation, a blinding method that has proved to be reliable previously [25, 26] as it induces the same skin sensations of active stimulation, namely, a mild tingling that usually fades away just after stimulation onset.

In addition, other methods to minimize blinding vulnerability will be employed, such as blinding raters to treatment applied, avoiding contact between subjects of different groups, and keeping the statistician unaware of treatment allocation during statistical analysis (i.e., “triple-blinded study”). Blinding is assessed at study endpoint by asking subjects to guess to which group they were assigned.

### 2.3. Study Design

This study is a randomized, double-blinded, sham-controlled trial in which 60 patients with treatment resistant bipolar depression are randomly assigned to two groups: active and sham tDCS. The patients who are using anticonvulsivants will be stratified at the randomization once since these drugs may change the results of the tDCS [27]. Subjects are followed for 6 weeks, and four assessments are performed: baseline, week 2, week 4, and endpoint (week 6). Adverse effects are assessed at week 2, week 4, and week 6 (endpoint); neuropsychological testing is conducted at baseline and endpoint. At the end of the trial, those who did not receive tDCS and did not respond (defined as ≥50% improvement in the HDRS-17) are offered an open-label phase of 10 daily sessions of tDCS.

Patients will return to the research center daily, for ten consecutive days and two additional returns. As this might be an issue for adherence (considering that São Paulo city has considerable traffic congestion) the patients are granted two nonconsecutive missing visits, which are replaced at the end of week 2. In fact, in our previous trials we observed that most patients present one or two absences during the acute treatment phase and that this did not impact tDCS antidepressant efficacy when the missing sessions are replaced at the end of the acute phase [28]. Dropout subjects are those who (1) miss three or more nonconsecutive visits or two consecutive visits during the initial 10-weekday stimulation period; (2) do not return at week 4 or week 6; (3) present serious clinical or psychiatric events during the trial, such as seizures or treatment-emergent (hypo)mania; (4) are lost to follow-up; (5) withdraw at their own request; (6) are excluded for safety reasons, including severe worsening of psychiatric condition and severe adverse effects. Missing data will be considered at random.

This study is already approved by the Hospitals Ethics Committee of the University Hospital of University of São Paulo, where the study is being conducted. The clinicaltrials.gov identifier is NCT02152878.

### 2.4. Procedures

Participants will be randomized according to a computer-generated list in http://www.randomization.com/. The allocation will be performed using opaque, sealed envelopes containing the code corresponding to the assigned group for each participant. This code will be imputed in the tDCS device that automatically delivers either a “sham” or “verum” stimulation, without awareness of the staff.

Participants will be assessed by training, certified psychiatrists, and/or clinical psychologists. Diagnosis will be confirmed using the MINI. The Hamilton Depression Rating Scale (HDRS), Montgomery-Asberg Depression Rating Scale (MADRS), Young Mania Rating Scale (YMRS), and Clinical Global Impression (CGI) will be applied at week 0 (baseline), week 2, week 4, and week 6 (endpoint). At baseline and endpoint we will also perform a comprehensive neuropsychological battery assessing cognitive domains such as attention, verbal fluency, working memory, and inhibitory control. The assessment of the clinical predictors of antidepressant response will be realized by the analysis of biomarkers, such as BDNF, interleukins, and genetic polymorphisms, as well as motor cortical excitability and heart rate variability, in addition to other clinical predictors of response like age, refractoriness, sex, type of bipolar disorder, type of drug class, chronicity, severity, and comorbidity with anxiety disorders. Adverse effects will be assessed using a standardized questionnaire [17].

The neuropsychological battery is described below.

(a) Verbal fluency: it is a neuropsychological test in which participants have to say as many words as possible from a category in a given time (60 seconds); it assesses executive function, language, phonemic fluency, and frontal areas associated with sustained attention, planning, organization, judgment/inhibitory control, strategy, and semantic perseveration [29].

(b) The stroop test: it measures the interference in the reaction time of a task, assessing executive function, attention, thought flexibility, selective attention, impulsivity, and resistance to interference. When the name of a color (e.g., “blue” or “green”) is printed in a color not denoted by the name (e.g., the word “green” printed in blue ink instead of green ink), naming the color of the word takes longer and is more prone to errors [30].

(c) The Rey Auditory-Verbal Learning Test (RAVLT): it asks the subject to recall a list of words that is read for five consecutive trials, followed by a recognition trial. The test assesses recent memory, verbal learning, susceptibility to interference, long memory recall (20 minutes), and recognition memory [31].

(d) Digit span (forward and backward): it evaluates attention, immediate memory (short-term) and verbal memory working memory, mental flexibility, concentration, and vigilance. In forward digit examinee repeats numbers sequence in same order as presented and in the backward in the reverse order [32].

This cognitive assessment is important because cognitive impairment is recognized as a feature of bipolar disorder and it is present in both acutely symptomatic and remitted states [33]. In fact, meta-analyses comparing remitted patients with bipolar disorder with healthy controls indicate that patients with bipolar disorder show moderate to large impairments on tests of attention, explicit memory, and processing speed and in different aspects of executive function [34–37].

In addition, we will assess several biomarkers. The importance of exploring them relies on identifying novel moderators and mediators of response, thus generating new data regardless of study results. Furthermore, identifying biological markers is important to accelerate translational research, using data from basic science in clinical practice and then backwards, addressing the clinical relevance of the findings in basic science.

(a) Brain derived neurotrophic factor (BDNF): BDNF is a neurotrophin related to neuronal survival and synaptic strengthening [38], roles that have raised the BDNF/neurotrophin hypothesis of depression that states the disorder is caused by low neuronal activity (and low BDNF levels) in some key areas and amelioration of symptoms as accompanied by restoration of normal brain activity (and normal BDNF levels). Decreased BDNF peripheral levels (i.e., serum or plasma) are a consistent finding in this area of research [39, 40]. The first report to find lower serum BDNF levels in bipolar disorder, during mania and depression, was published by Cunha et al. in 2006 and was quickly replicated [41]. Machado-Vieira et al. [42] reported decreased plasma BDNF levels in unmedicated patients with bipolar disorder during a manic episode. de Oliveira et al. [43] found decreased levels of BDNF in manic and depressive patients regardless of the medication status. One study reported significantly decreased mRNA levels of lymphocyte-derived BDNF and decreased protein BDNF levels in platelets in manic unmedicated children and adolescents versus controls [44]. Two independent longitudinal studies conducted with manic patients found that peripheral BDNF levels increase after successful pharmacological treatment [45, 46]. Intriguingly, however, the two tDCS trials that assessed BDNF blood levels did not observe BDNF increasing after treatment [47, 48]. Thus, we will explore whether BDNF increases after depression treatment, contributing to the understanding of some mechanisms of action of this new neuromodulatory technique.

(b) Heart rate variability (HRV): recent studies and meta-analyses substantiate that depressed patients present decreased HRV; however, antidepressant treatment also seems to decrease HRV as well [49, 50]. In our SELECT-TDCS trial, we did not observe that either tDCS or sertraline changed HRV levels, which were lower compared to matched, healthy controls [51]. In this study, we will be able to assess whether HRV values change after tDCS in bipolar depression.

(c) Peripheral inflammatory-biomarkers: a number of studies suggest that there may be a link between inflammation and neuroplasticity pathways. With respect to inflammation, a number of studies have also found that depressive and to a greater degree manic states are associated with increased peripheral levels of proinflammatory cytokines, such as interleukin- (IL-) 2, IL-6, IL-8, and tumor necrosis factor- (TNF-) *α* [52–54]. It has been demonstrated that manic patients with BD have increased IL-6 and TNF-*α* protein [54] and mRNA levels when compared to healthy controls. In a recent study, it was showed that patients with bipolar II disorder had significantly lower levels of sTNF-R1 than the patients with bipolar I disorder and patients in a depressive state had significantly lower levels of sTNF-R1 than the patients in manic/hypomanic and euthymic states [55]. In addition, studies have shown that tDCS can improve the cognitive impairments in bipolar depression and that this may be related to serum levels of some biomarkers. Bauer et al. showed that high levels of peripheral inflammatory-cytokine reduced brain derived neurotrophic factor (BDNF) levels were associated with poor cognitive performance in bipolar disorder [56]. In the first study assessing inflammatory cytokines in tDCS, we found that their levels decrease over time, but also in the placebo group, suggesting a nonspecific placebo effect [57].

(d) Genetic biomarkers: consistent results and significant meta-analyses reported association between polymorphisms located in the genes encoding BDNF, COMT, and 5-HTT and BD; such associations were also found for other psychiatric disorders such as schizophrenia, unipolar depression, and eating disorders [58]. No hypothesis-driven strategies based on the whole genome exploration were thus developed in order to identify relevant genetic biomarkers of BD. In particular, recent technological improvement allowed genome-wide association studies (GWAS) of large groups of patients and controls. These strategies consist of comparing allele frequencies between patients and controls for thousands of single nucleotide polymorphisms (SNPs) spanning the genome. Meta-analyses of these GWAS have been conducted and several candidate risk loci in BD have been identified, for instance, in* CACNA1C* (alpha 1C subunit of the L-type voltage-gated calcium channel) and* ANK3* (ankyrin 3) [59–64]. In the present study we are going to collect DNA to assess whether the genetic polymorphisms 5-HTTLPR, COMT, ANK3, CACNA1C, and BDNF predict antidepressant tDCS response in BD, analogously to our SELECT-TDCS trial, in which we found that the 5-HTTLPR polymorphism (but not Val66Met BDNF) moderates tDCS response in MDD [47].

(e) Motor cortical excitability: this assessment is performed using a transcranial magnetic stimulation (TMS) device that triggers simple or paired pulses. Motor evoked potentials (MEPs) are recorded using an electromyography (EMG). Motor cortical excitability for tDCS is used since the early reintroduction of studies with tDCS by Nitsche and Paulus [18] in 2000, who used a TMS/EMG system for measuring motor evoked potentials (MEPs) before and after anodic or cathodic stimulation. Through this procedure, the authors found that an electric current of low intensity generated over the motor cortex had polarity-dependent effects (i.e., the anode led to higher MEPs amplitudes and cathode at lower MEPs amplitudes, after the stimulation), intensity dependent and late effects, that remained after the end of tDCS. Even today, a line of very important research involves the measurement of the motor cortex excitability after use of tDCS in combination with psychoactive drugs (see, e.g., [65]).

In patients with a bipolar affective disorder, cortical hyperexcitability, reflected by reduced cortical silent period, short-interval intracortical inhibition, and interhemispheric inhibition, was shown in one study [66]. Other studies show variable results in depressive and bipolar disorder, making definitive conclusions difficult. In our study, we will explore cortical excitability parameters at different time points, evaluating whether they are predictors and markers of clinical improvement.

### 2.5. Statistical Analysis

The primary analysis will be a repeated-measures analysis of variance with tDCS (2 levels: active and sham) as the between-independent variable and time (4 levels: weeks 0, 2, 4, and 6) as the within-independent variable. HDRS is the dependent variable. Our hypothesis is that the interaction of time with tDCS will be significant, with active tDCS being superior to sham tDCS at week 6. We will also explore secondary outcomes using the MADRS as the dependent variable. In addition, we will perform multivariate logistic regressions having response (decrease in HDRS scores > 50%) and remission (HDRS < 8) as dependent variables. Frequency of treatment-emergent (hypo)mania (YMRS > 8) will be compared among groups using Fisher's exact test or the chi-square test.

The primary analysis will be an intention-to-treat (ITT) analysis, handling missing data using the last observation carried forward (LOCF) approach. We will also perform per protocol (PP) analyses. The sample size was calculated based on Kalu et al. meta-analysis [24] and the results of our study in unipolar depression [19], considering a power of 80% and a two-tailed *p* of 5%, and also taking into account an expected attrition rate of 10–15% as observed in our previous MDD trial.

## 3. Results from the Pilot Study Phase

In the pilot phase of our study, we assessed methodological issues and other relevant topics for the quality of the study, such as the adherence to the project and standardization of questionnaires.

In this phase we enrolled 5 patients (4 females/1 male, aged 23–49 years) with bipolar depression type I or II who presented treatment failure to at least two previous treatments. Although all enrolled patients received active stimulation, they were all double-blindly assessed (i.e., patients and raters were unaware of the treatment applied), as they were stimulated in the same research center where other ongoing tDCS trials are being carried out.

**Figure 1:**
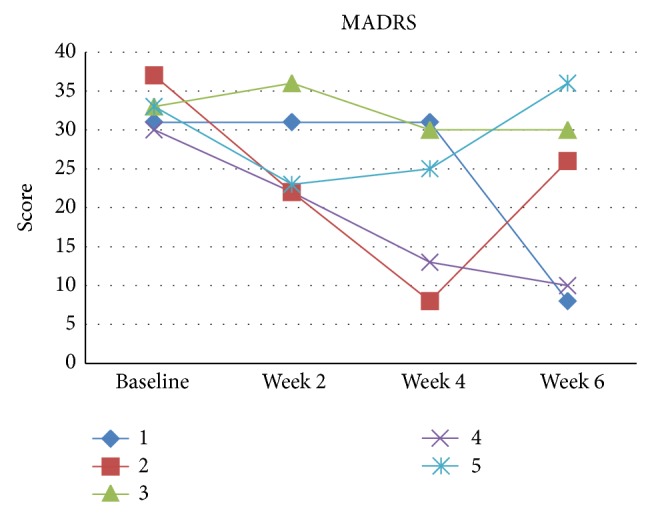
MADRS scores for each patient (pilot phase).

**Figure 2:**
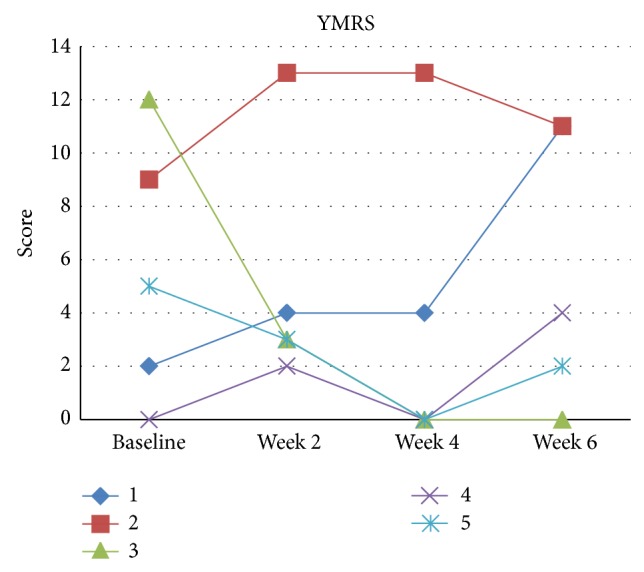
YMRS scores for each patient (pilot phase).

**Figure 3:**
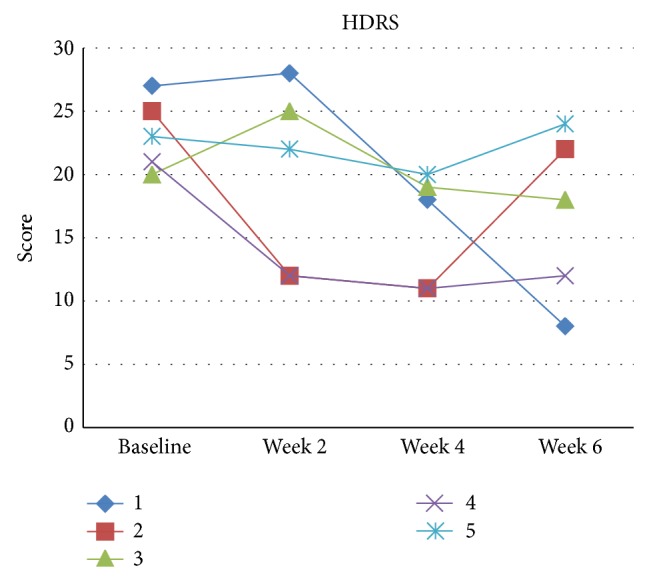
HDRS scores for each patient (pilot phase).

Sample characteristics are described in Table 1. Two patients presented clinical response, two presented partial response, and one showed no improvement (Table 2 and Figures 1, 2, and 3). All patients tolerated tDCS well without any serious adverse effects. In this pilot phase, we followed the same procedures as described in Section 2, with two main exceptions: in the pilot phase we were not able to perform, due to technical reasons, two extra tDCS sessions at weeks 4 and 6; that is, participants of the pilot phase received only 10 tDCS sessions; and during the pilot phase the YMRS cut-off was 12 (and not eight).

### 3.1. Case 1

Case 1 was a bipolar depression type II, 24-year-old woman. The depressive episode was of moderate to severe severity without psychotic symptoms. She had failed several antidepressant and mood stabilizers. At study entry, she was on lamotrigine 300 mg/day, topiramate 75 mg/day, risperidone 1 mg/day, and alprazolam 0,25 mg/day. During tDCS, drug treatment was not altered. Her HDRS score at baseline and endpoint was 27 and 8, respectively. However, her YRDS score increased from 2 to 11.

### 3.2. Case 2

Case 2 was a 49-year-old man with bipolar depression type II. The depressive episode was severe and he presented failure to several drug treatments, including lithium, sertraline, and quetiapine. During tDCS treatment, the patient improved at week 2 and week 4, although he presented recrudescence of symptoms at endpoint. The score on YMRS at beginning was higher than 8, and he maintained manic symptoms at endpoint.

### 3.3. Case 3

Case 3 was a 48-year-old woman with bipolar depression type I. The depressive episode was of moderate severity. She had failed two antidepressant trials and one mood stabilizer treatment of adequate dose and duration, and at study entry she was drug-free. She did not present improvement of depressive symptoms (baseline and endpoint HDRS scores of 20 and 18, resp.).

### 3.4. Case 4

Case 4 was a 30-year-old woman with bipolar depression type II. The depressive episode was of severe severity and she had failed to respond to at least two antidepressants trials of adequate dose and duration. When she entered the study, she was using quetiapine 350 mg/day, topiramate 75 mg/day, sodium valproate 1000 mg/day, and clonazepam 2 mg/day. She presented clinical response, as the HDRS changed from 21 to 12 from baseline to endpoint.

### 3.5. Case 5

Case 5 was a 44-year-old woman with a bipolar depression type I. The depressive episode was of moderate-to-severe severity. When she enrolled into the study, she was using venlafaxine 75 mg/day, topiramate 50 mg/day, and levomepromazine 25 mg/day. She did not present depression improvement, as her endpoint score was 24.

## 4. Discussion

The BETTER study will be one of the largest trials to date assessing the efficacy of tDCS specifically in bipolar depression. The sample size was adequately powered to minimize type I and type II errors and to handle a study attrition of up to 15%. We will also enroll patients with type I and type II bipolar depression, of different degree of refractoriness and also enrolling anxiety disorders as a comorbidity, which will enhance external generalizability of tDCS in different contexts. Our treatment protocol consists in using 2 mA for 30 min daily per ten consecutive workdays plus two extra sessions every other week, similarly as applied in Brunoni et al. [19] study for major depression (although less applied in Loo et al. study [21], which used 15 tDCS sessions). Additionally, in our study we use a bilateral frontal stimulation (anode on F3 and cathode on F4)—similar to Brunoni et al. [22] but different from others that used a cathode on the contralateral supraorbital prefrontal cortex. Theoretically, this could be more advantageous in modulating left/right imbalance observed in major depression [67] given that DLPFC is a critical area as shown by other noninvasive brain stimulation studies [68]. We will be able to assess, therefore, the efficacy of this montage specifically for bipolar depression.

Importantly, our trial duration (6 weeks) could be considered relatively short as symptoms might improve over 2-3 months. Nonetheless, a consensus of trialists for depression treatment considered that current evidence favors shortening new antidepressant trials to 3 to 4 weeks from the current 6 to 8 [69], as this smaller timeframe is suitable to differentiate between active and placebo effects.

### 4.1. Results of the Pilot Phase

The results of the pilot phase are also of value, although limited by the small sample. All patients received active tDCS; however, all of them were assessed in a double-blinded fashion (neither patients nor raters were aware of the treatment applied), since the same raters of other ongoing sham-controlled tDCS trials evaluated them. This preliminary data allowed us to observe that 2 of 5 (40%) patients presented clinical response and 1 of 5 (20%) remitted and an overall improvement (change in depression scores) of approximately 30%, similar rates to what was observed in most unipolar tDCS trials [28]. Importantly, in the pilot phase we were not able to perform two extra tDCS sessions after the acute treatment period. Although it is still unknown whether extra tDCS sessions are associated with greater improvement, in a recent meta-analysis we found that there is at least a trend (*P* = 0.1) for such association. Therefore, in the present study we will perform 12 tDCS sessions, a similar amount of sessions of our unipolar depression study, in which we were able to detect a superior improvement of active versus sham tDCS.

Another important aspect is that we did not observe cognitive worsening throughout the trial, as the cognitive performance in a wide array of neuropsychological tests remained utterly unchanged. This was also observed in unipolar depression trials, adding more data regarding the absence of cognitive adverse effects related to tDCS.

Finally, in the pilot study phase we assessed manic symptoms. We observed that both patients who presented YMRS > 8 at baseline did not present depression improvement and, in addition, one of them maintained his manic symptoms. This could have occurred due to relatively liberal criteria (YMRS < 12) that we used in the pilot phase. Also based on these findings, we opted to use a stricter (YMRS < 8) eligibility criterion, to exclude depressive episodes with mixed features that could create a bias in the study towards nonresponsiveness. Moreover, one of the patients with a baseline YMRS of 2 had a final score of 11. Although these findings cannot be generalized, as they are limited due to the small sample size, there are other reports in the literature associating tDCS with treatment-emergent mania in unipolar and bipolar depression [19, 70–73]. In this regard, the present study will aid to investigate whether tDCS induces manic symptom in patients with a bipolar diathesis.

## 5. Conclusion

The BETTER study will address the efficacy of transcranial direct current stimulation for bipolar disorder patients with treatment-resistant depression using a randomized, sham-controlled design. The investigation of the relationship of biological markers with depression response will also contribute in understanding the pathophysiology of bipolar depression as well as the mechanisms of action of this new technique of neuromodulation. Therefore, our trial can generate important findings in the fields of clinical treatment of bipolar depression and noninvasive brain stimulation.

## Figures and Tables

**Table 1:** Clinical and demographic characteristics of the sample (pilot phase).

Sample	Data
Subjects	5
Age, mean (SD)	39 (11.31)
Gender, *n* (%)	
Male	1 (20)
Female	4 (80)
Pharmacotherapy	
*Antidepressant drug*, *n* (%)	1 (20)
Mood stabilizer, *n* (%)	3 (60)
Antipsychotic, *n* (%)	3 (60)

**Table 2:** Changes in depressive symptoms throughout the study (pilot phase).

	Baseline	Week 2		Week 4		Week 6	
	Mean (SD)	Mean (SD)	% (SD)	Mean (SD)	% (SD)	Mean (SD)	% (SD)
MADRS	32.8 (2.68)	26.8 (6.37)	17.68 (21.16)	21.4 (10.35)	33.67 (32.99)	22 (12.40)	34.11 (35.97)
HDRS	23.2 (2.86)	19.8 (7.42)	14.10 (32.42)	15.8 (4.43)	30.99 (21.82)	16.8 (6.72)	26.17 (30.10)
HAMA	32 (6)	24.6 (5.54)	21.51 (20.14)	20.4 (9.01)	37.59 (19.00)	22.8 (12.27)	29.56 (30.03)
Response (%)			0		2 (40)		2 (40)
Remission (%)			0		0 (0)		1 (20)

MADRS: Montgomery-Asberg Depression Rating Scale; HDRS: Hamilton Depression Rating Scale; HAMA*: *Hamilton Anxiety Rating Scale; YMRS: Young Mania Rating Scale; percentage represents percentage of change, calculated as ((score at time point − score at baseline)/score at baseline) ∗ 100. Clinical response was defined as >50% HDRS-17 score improvements from the baseline and the remission was defined as HDRS-17 score <8.
